# The first complete mitochondrial genome of *Stomopneustes variolaris* (Lamarck, 1816) from the Stomechinidae (Echinoidea: Stomopneustoida)

**DOI:** 10.1080/23802359.2021.1947918

**Published:** 2021-07-06

**Authors:** Shengping Zhong, Longyan Zhao, Lianghua Huang, Yonghong Liu, Guoqiang Huang

**Affiliations:** a Institute of Marine Drugs, Guangxi University of Chinese Medicine, Nanning, China; b Guangxi Engineering Technology Research Center for Marine Aquaculture, Guangxi Institute of Oceanology Co., Ltd, Beihai, China

**Keywords:** Mitochondrial genome, *Stomopneustes variolaris*, Stomopneustoida

## Abstract

Sea urchins (Echinoidea) are key components of marine benthic communities and many are commercially important fishery resources as luxury and healthy seafood. However, despite their high ecological and economic value, the mitochondrial genomes of all sea urchins have yet to be analyzed. In this study, we report the first complete mitochondrial genome of Stomopneustidae from *Stomopneustes variolaris*. The mitogenome has 15,767 base pairs (59.77% A + T content) and contains 37 genes (13 protein-coding, 22 transfer RNAs and 2 ribosomal RNAs), plus a putative control region. This study provides useful molecular resources for clarifying evolutionary and phylogenetic histories of sea urchins.

Sea urchins also known as echinoids, are the most important and conspicuous components of local marine benthic ecosystems (Kroh and Smith 2010). The class Echinoidea contains more than 850 living species distributed worldwide, many of which are ecologically and economically important due to their abundance in benthic ecosystems (Mongiardino Koch et al. 2018). One such species, *Stomopneustes variolaris* is regarded as a ‘healthy’ seafood for its valuable nutrition and healthy chemical compounds (Archana and Babu 2016; Francis and Chakraborty 2020). Although the classification of echinoids had been elucidated using morphological data, the phylogenetic status and evolutionary history of echinoids has become challenged, especially relying on only morphology-based analyses (Mongiardino Koch et al. 2018). Molecular data have been shown to be a useful tool for understanding phylogenetic relationship of sea urchins (Kroh and Smith 2010; Mongiardino Koch et al. 2018). Unfortunately, up to now, published complete mitogenome sequences of echinoids are limited, and the mitochondrial genomes of a member of the Stomopneustidae has not been sequenced. Here, we report the first complete mitochondrial genome of an echinoid classified to the Stomopneustidae, which will provide useful molecular data for phylogenetic and evolutionary analyses of sea urchins.

Tissue samples of *S. variolaris* from five individuals were collected from HaiNan province, China (QiongHai, 19.192971 N, 110.609444 E) by local diving fishermen, together with the whole body and DNA specimen (#JP0325) were deposited at the Marine Biological Museum, Guangxi Institute of Oceanology, Beihai, China (http://www.gxas.cn/kypt/kxpj/kpcg, Shengping Zhong, shpzhong@foxmail.com). The total genomic DNA was extracted from the muscle of Aristotle’s lantern using an SQ Tissue DNA Kit (OMEGA, Guangzhou, China) following the manufacturer’s protocol. DNA libraries (350 bp insert) were constructed with the TruSeq NanoTM kit (Illumina, San Diego, CA) and were sequenced (2 × 150bp paired-end) using the HiSeq platform at BGI Company, China. Mitogenome assembly was performed by MITObim (Hahn et al. 2013). The cytochrome oxidase subunit I (COI) gene of *S. variolaris* (GenBank accession number: AF279195) was chosen as the initial reference sequence for the MITObim assembly. Gene annotation was performed by MITOS (http://mitos2.bioinf.uni-leipzig.de).

The complete mitogenome of *S. variolaris* (GenBank accession number: MW147664) is 15,767 bp in length and is similar to other published echinoids (Lin et al. 2020). The mitogenome contains a conserved set of 13 protein-coding genes (PCGs), 2 ribosomal RNA genes, 22 transfer RNA genes, and a putative control region. A total of 37 genes were annotated and 164 nucleotides represent the putative control region. The overall base composition of the mitogenome is estimated to be A 29.65%, T 30.12%, C 23.54% and G 16.69%, with a high A + T content of 59.77%, which is most similar to *Glyptocidaris crenularis* (58.96%) (Ding et al. 2017). The phylogenetic tree of 35 echinoderms was constructed by maximum-likelihood method via PhyML online server (http://www.atgc-montpellier.fr/phyml/), using GTR substitution model with 100 bootstrap replicates. The phylogenetic analysis inferred from the concatenated nucleotides sequences of 13 PCGs fully resolved *S. variolaris* on a branch with *G. crenularis* in the order Stomopneustoida sister in position to the Arbacioida echinoids (Figure 1). These findings are consistent with the phylogenetic analyses of echinoids using mitogenome sequences (Lin et al. 2020) and skeletal-based characters (Kroh and Smith 2010). The complete mitochondrial genome sequence of *S. variolaris* will be useful for better resolving the phylogenetic and evolutionary history of sea urchins.

**Figure 1.**
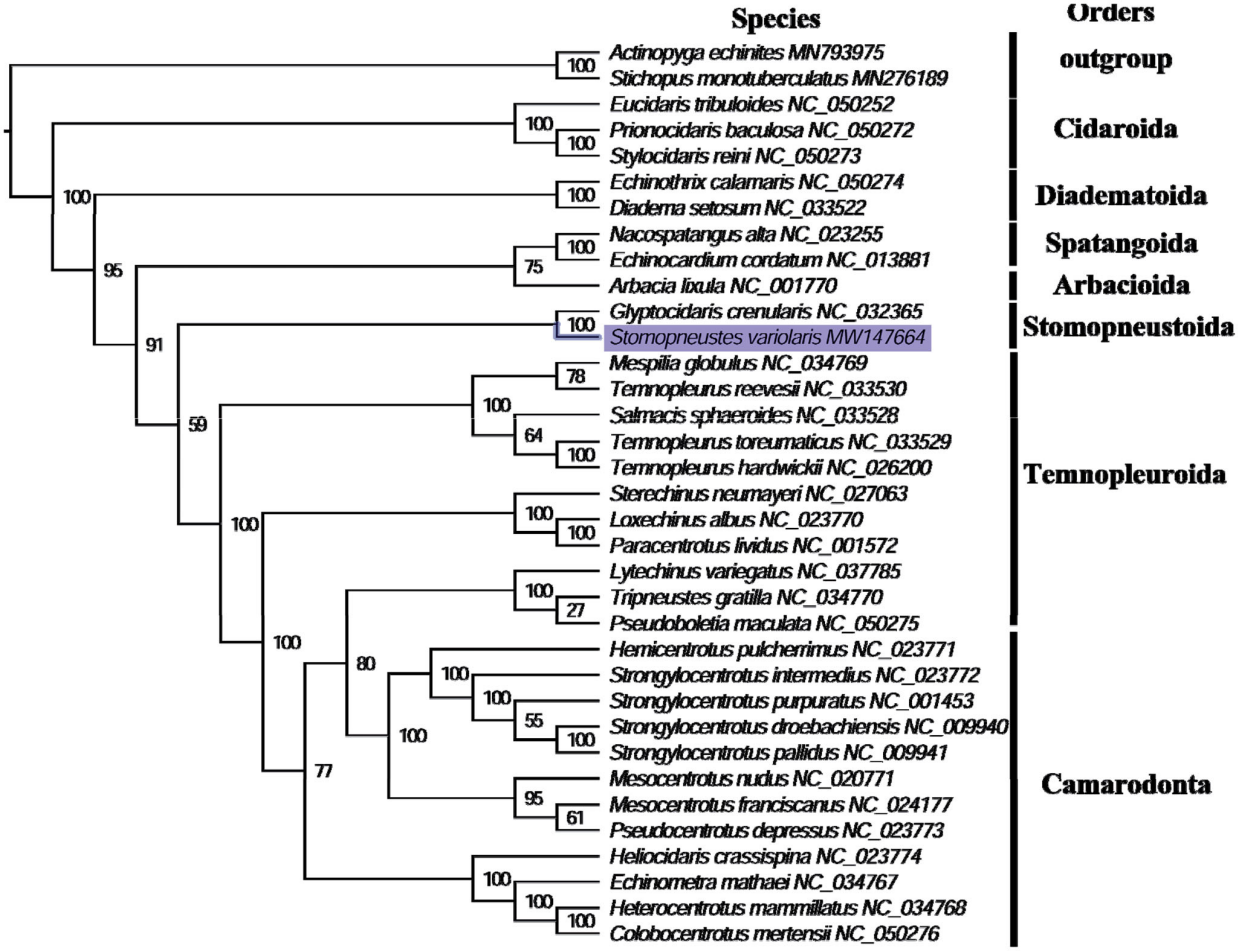
Phylogenetic tree of 35 species in echinoderms. The complete mitogenomes were downloaded from GenBank and the phylogenic tree based on the concatenated nucleotide sequences of 13 mitochondrial PCGs was constructed by maximum-likelihood method with 100 bootstrap replicates. The bootstrap values were labeled at each branch nodes, holothuroids (*Actinopyga echinites* and *Stichopus monotuberculatus*) were chosen to be outgroup species.

## Data Availability

The genome sequence data that support the findings of this study are openly available in GenBank of NCBI at (https://www.ncbi.nlm.nih.gov/) under the accession no. MW147664. The associated BioProject, SRA, and Bio-Sample numbers are PRJNA729857, SRR14535489, and SAMN19184015, respectively.
